# Cardiac arrest due to tamponade during secondary-stage endovascular stent implantation in a patient with DeBakey type I dissection: a case report and literature review

**DOI:** 10.3389/fmed.2026.1815531

**Published:** 2026-04-28

**Authors:** Hongyang Chen, Tao Zhu

**Affiliations:** 1 Department of Anesthesiology, West China Hospital, Sichuan University, Chengdu, Sichuan, China; 2 The Research Units of West China (2018RU012)-Chinese Academy of Medical Sciences, West China Hospital, Sichuan University, Chengdu, Sichuan, China; 3 Department of Anesthesia and Surgery, Xizang Hospital of West China Hospital, Sichuan University, Chengdu, China

**Keywords:** aortic dissection, cardiac arrest, endovascular stent grafting, protamine reaction, staged TEVAR, tamponade

## Abstract

**Background:**

Hemorrhagic cardiac tamponade is a rare but potentially fatal complication of endovascular aortic intervention, mostly caused by aortic rupture or cardiac perforation; cases without arterial/cardiac injury are extremely uncommon. Protamine anaphylactic reaction is a common perioperative adverse event, and its synergistic effect with cardiac tamponade on hemodynamic instability is often overlooked in clinical practice.

**Case presentation:**

We report a 63-year-old man with 10 years of poorly controlled hypertension and a 2-year history of stroke who was diagnosed with DeBakey type I aortic dissection. The patient underwent hemi-arch replacement combined with Bentall operation as the first-stage surgery, and the second-stage descending aorta stent implantation (femoral artery retrograde implantation, chimney stent placement in the innominate artery, and carotid-carotid cross-over bypass) was performed on the 20th postoperative day in line with clinical consensus. During the second-stage surgery, protamine anaphylaxis occurred first, followed by progressive hemodynamic instability; the patient then suffered cardiac arrest due to acute cardiac tamponade. Transesophageal echocardiography (TEE) confirmed a large amount of pericardial effusion with right heart chamber collapse, and subxiphoid surgical pericardial window and drainage were immediately performed, with 300 mL of bloody fluid aspirated. Anticoagulant/antiplatelet therapy was timely adjusted after drainage, and the patient was discharged without neurological complications 2 weeks later. Serial perioperative biological and physiological data were comprehensively monitored and recorded during the whole process.

**Conclusion:**

Cardiac tamponade can be readily reversed with timely recognition and intervention, and TEE is the gold standard for its rapid perioperative diagnosis. For patients undergoing staged TEVAR for DeBakey type I aortic dissection with a history of cardiac surgery, long procedural duration, and systemic anticoagulation, tamponade should be highly vigilant even without obvious aortic/cardiac injury. The protamine reaction can synergize with tamponade to aggravate hemodynamic disorder and mask its early manifestations, requiring enhanced multi-modal monitoring. Timely subxiphoid surgical pericardial window drainage is an effective intervention for tamponade-induced cardiac arrest, and individualized adjustment of anticoagulation therapy is crucial for avoiding rebleeding.

## Introduction

In the past decades, hybrid surgical techniques have been widely adopted for the treatment of aortic dissection and have achieved excellent clinical outcomes (1, 2, 28). Staged thoracic endovascular aortic repair (TEVAR) is the main hybrid strategy for DeBakey type I aortic dissection, which can balance the surgical risk and the risk of dissection progression (3, 4). At present, clinical consensus recommends performing staged TEVAR 2–4 weeks after the initial ascending aortic repair for hemodynamically stable patients, and there is no unified international or domestic guideline defining the optimal timing (5).

This hybrid technique is associated with a variety of complications, including aortic rupture, perforation, endoleak, paraplegia, stroke, and acute ascending aortic dissection (6, 7). Hemorrhagic cardiac tamponade is a rare but fatal complication with an overall incidence of <1% in relevant cohorts, usually caused by aortic perforation or cardiac rupture (8, 9). Cases of cardiac tamponade without arterial/cardiac injury during staged TEVAR are extremely rare, and their etiology and clinical management lack sufficient clinical evidence and discussion (10, 11).

Protamine anaphylactic reaction is a common perioperative adverse event in vascular surgery with systemic anticoagulation, which can cause acute hemodynamic instability, pulmonary vasoconstriction, and right ventricular failure (12). However, the synergistic effect of protamine reaction and cardiac tamponade on hemodynamic disorder, as well as its interference with the early diagnosis of tamponade, has not been fully elucidated in previous studies.

Herein, we report a case of cardiac arrest due to acute cardiac tamponade without aortic/cardiac injury in a patient undergoing second-stage TEVAR for DeBakey type I aortic dissection after protamine anaphylactic reaction. We conduct a rigorous etiological analysis of tamponade, discuss the correlation between protamine reaction and tamponade, and supplement this with a systematic literature review on pericardial tamponade complicating endovascular aortic procedures. This case provides valuable clinical references for perioperative monitoring, early diagnosis, and individualized treatment of severe complications in staged aortic dissection surgery.

## Case presentation

**Figure 1:**
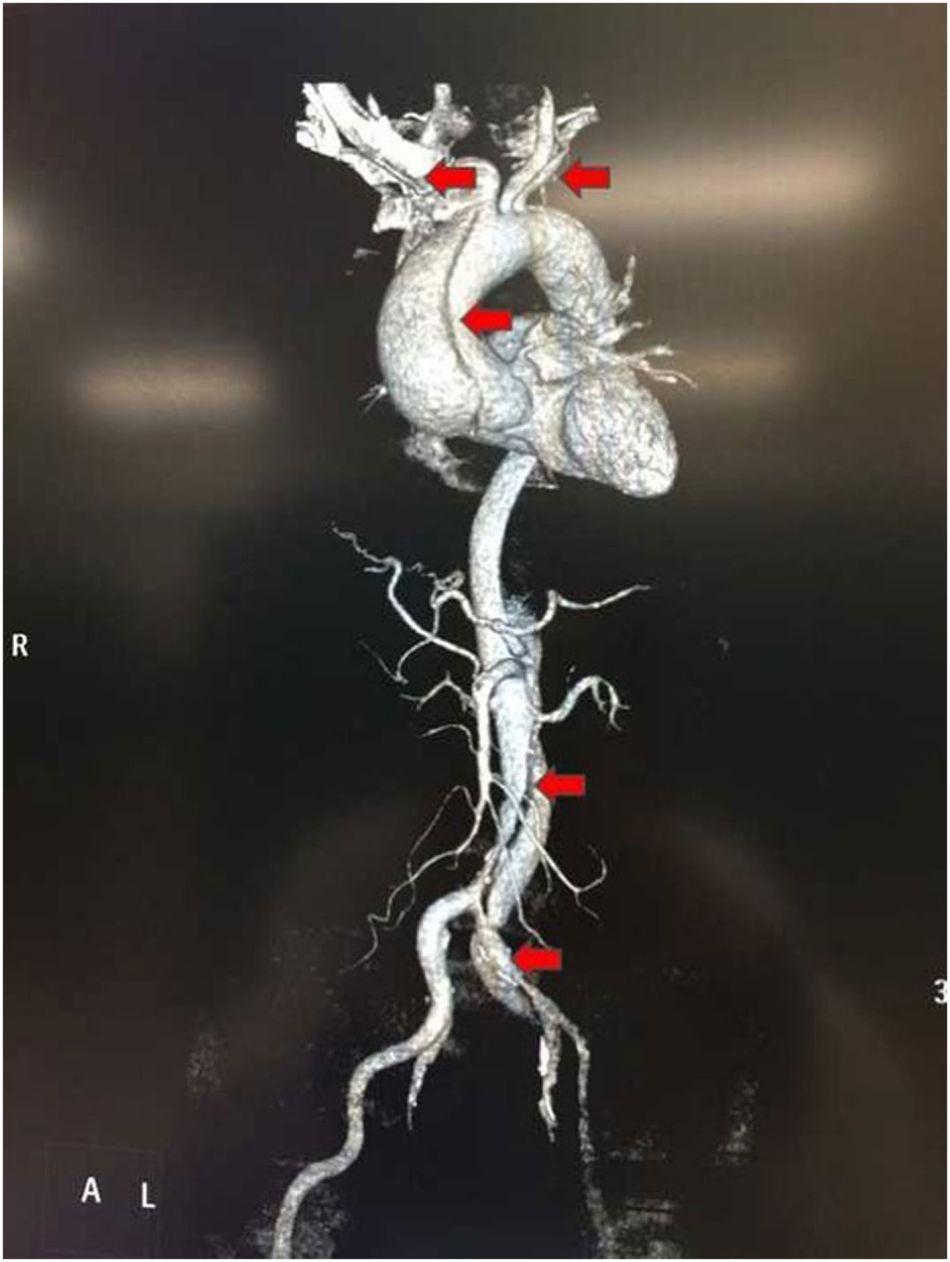
Reconstructed image of computed tomography angiography (CTA). Arrows indicate the dissection of the aortic wall and the false lumen of the DeBakey type I aortic dissection extending from the aortic root to the left external iliac artery.

A 63-year-old man presented to the emergency room with persistent chest pain for 3 days. Computed tomography angiography (CTA) indicated DeBakey type I aortic dissection extending from the aortic root to the left external iliac artery, involving the brachiocephalic trunk and left subclavian artery (Figure 1). Preoperative CTA and transesophageal echocardiography (TEE) measured the aortic root diameter as 52 mm; transthoracic echocardiography (TTE) confirmed the diagnosis and identified moderate aortic valve regurgitation. The exact mechanism of regurgitation confirmed by intraoperative TEE was annular dilatation caused by aortic dissection and interference of the dissection flap with aortic valve leaflet coaptation. No pericardial effusion (trace, mild, moderate, or severe) was found on all initial CTA and TTE examinations. The patient’s medical history included 10 years of poorly controlled hypertension (systolic blood pressure sustained at 160–180 mmHg) and a neurologic stroke 2 years prior with no residual neurological deficits.

### First-stage surgical decision and implementation

Given the patient’s pathological characteristics, valve-sparing root replacement (David procedure) was not performed because the aortic dissection extensively involved the aortic valve leaflets and annulus, and the acute pathological state of the patient made the Bentall procedure the first choice to ensure surgical safety and complete anatomical repair (27). Hemi-arch replacement and Bentall operation were performed under cardiopulmonary bypass after admission. The hemi-arch replacement included the innominate artery and left common carotid artery in the repair range, and the left subclavian artery was preserved due to no obvious dissection involvement and intact vascular anatomy. The primary entry tear of the aortic dissection was located in the ascending aorta 15 mm above the aortic valve annulus, which was completely repaired by vascular replacement during the first-stage operation. Postoperative imaging evaluation confirmed that the true lumen of the residual arch was patent, the false lumen was partially thrombosed, and no obvious dissection progression was found.

The patient had no clinical evidence of active bleeding during the postoperative monitoring period, including no persistent increase in mediastinal drainage volume, no decline in hemoglobin/hematocrit, and no hemodynamic instability caused by bleeding. The mediastinal drainage tubes were removed on the seventh postoperative day in accordance with the standard clinical criteria for cardiac surgery in our center: (1) the total 24-h mediastinal drainage volume was less than 100 mL and showed a continuous decreasing trend; (2) no evidence of hemothorax, cardiac tamponade, or other bleeding-related complications on bedside TTE; and (3) stable hemodynamics and normal coagulation function of the patient.

### Interval imaging evaluation (day 0–20 post first-stage surgery)

Contrast-enhanced CTA of the aorta was performed on the 14th postoperative day, considering the patient’s stable vital signs, no obvious clinical symptoms, and no contrast agent-related contraindications (normal renal function, no iodine allergy). The CTA results showed: (1) the true lumen of the ascending aorta and proximal arch was fully patent, the false lumen was partially thrombosed with no obvious expansion; (2) the false lumen of the descending aorta to the iliac artery was still patent but no new/enlarging entry tears, impending rupture signs or dissection progression were found; (3) bilateral femoral arteries were patent with intact vascular walls, and the access route was confirmed to pass through the true lumen by CTA and preoperative vascular ultrasound, with no false lumen puncture risk, which was the basis for selecting the femoral artery as the endovascular access for the second-stage surgery. No dedicated TEE/TTE was performed during the interval, and routine clinical vital sign monitoring, laboratory tests, and physical examinations were conducted.

### Second-stage surgical procedure

The second-stage surgery for aortic stent implantation and supra-aortic debranching was scheduled on the 20th postoperative day. The patient’s initial vital signs were stable (blood pressure 135/82 mmHg, heart rate 78 bpm, SpO_2_ 98% in room air), and general anesthesia was induced successfully with baseline near-infrared spectroscopy (NIRS) of 61% on both sides. Systemic anticoagulation for staged TEVAR was achieved with unfractionated heparin 150 IU/kg, and activated clotting time (ACT) was maintained at 375–999 s (the standard anticoagulation target for endovascular aortic intervention in our center). Under the guidance of digital subtraction angiography (DSA), an aortic stent was implanted retrogradely via the right femoral artery, covering from the innominate artery to the proximal descending aorta. A chimney stent was placed in the innominate artery to maintain blood supply, and a carotid-carotid cross-over bypass was performed to ensure cerebral perfusion. The entire procedural duration was 7 h, with an hourly urine output of 0.5–1.0 mL/kg during the operation and a perioperative core body temperature maintained at 36.0–37.2 °C (Table 1).

**Table 1:** Serial perioperative clinical and biological data of the patient.

Time point	BP (mmHg)	CVP (mmHg)	HR (bpm)/Rhythm	Lactate (mmol/L)	Hb/Hct (g/L/%)	pH/pO_2_/pCO_2_ (mmHg)	BE (mmol/L)	INR	Fibrinogen (g/L)	ACT (s)
Pre-anesthesia	135/82	8	78/SR	1.2	132/39	7.42/98/35	0.5	1.02	3.5	125
Anticoagulation completed	128/76	10	85/SR	1.3	130/38	7.40/300/38	–1.0	–	3.4	420
Protamine reaction	70/40	15	125/Af	2.1	128/37	7.35/280/42	–3.5	–	3.2	180
Pre-cardiac arrest	95/55	19	130/Af	3.5	125/36	7.30/250/45	–5.0	1.10	3.0	200
Post-cardiac arrest (ROSC)	145/93	30	145/Af	4.2	122/35	7.25/220/48	–7.5	1.15	2.8	210
Post-drainage (3 min)	147/74	24	98/SR	3.8	120/34	7.32/260/42	–4.0	1.12	2.9	250
Post-drainage (24 h)	125/70	12	82/SR	1.8	123/35	7.40/300/36	–0.5	1.05	3.2	130
Discharge	130/75	9	76/SR	1.1	135/40	7.43/97/35	0.8	1.01	3.6	128

BP, blood pressure; CVP, central venous pressure; HR, heart rate; SR, sinus rhythm; Af, atrial fibrillation; ROSC, return of spontaneous circulation; Hb, hemoglobin; Hct, hematocrit; BE, base excess; INR, international normalized ratio; ACT, activated clotting time; −, not detected.

### Perioperative adverse events and management

The patient’s vital signs remained stable until protamine was administered for heparin reversal, which immediately induced anaphylactic shock (blood pressure dropped to 70/40 mmHg, heart rate increased to 125 bpm, and SpO_2_ 92%). Epinephrine (100 μg) was intravenously administered immediately, and the hemodynamic status improved but was complicated by new-onset atrial fibrillation; only intravenous amiodarone was used for rhythm control, and no oral anticoagulants were administered at this stage.

Shortly after the protamine reaction, norepinephrine was required for continuous infusion to maintain blood pressure, and the central venous pressure (CVP) increased from 17 to 19 mmHg; the right NIRS decreased to 35%, and the left NIRS was undetectable. Before cardiac tamponade occurred, the patient had not yet started oral antiplatelet therapy (the standard regimen of aspirin + clopidogrel for aortic stent grafts is usually initiated 24 h after the operation in our center for hemodynamically stable patients). DSA reexamination confirmed that the stent position was normal, the branch blood flow was unobstructed, and no aortic rupture/perforation was found.

**Figure 2:**
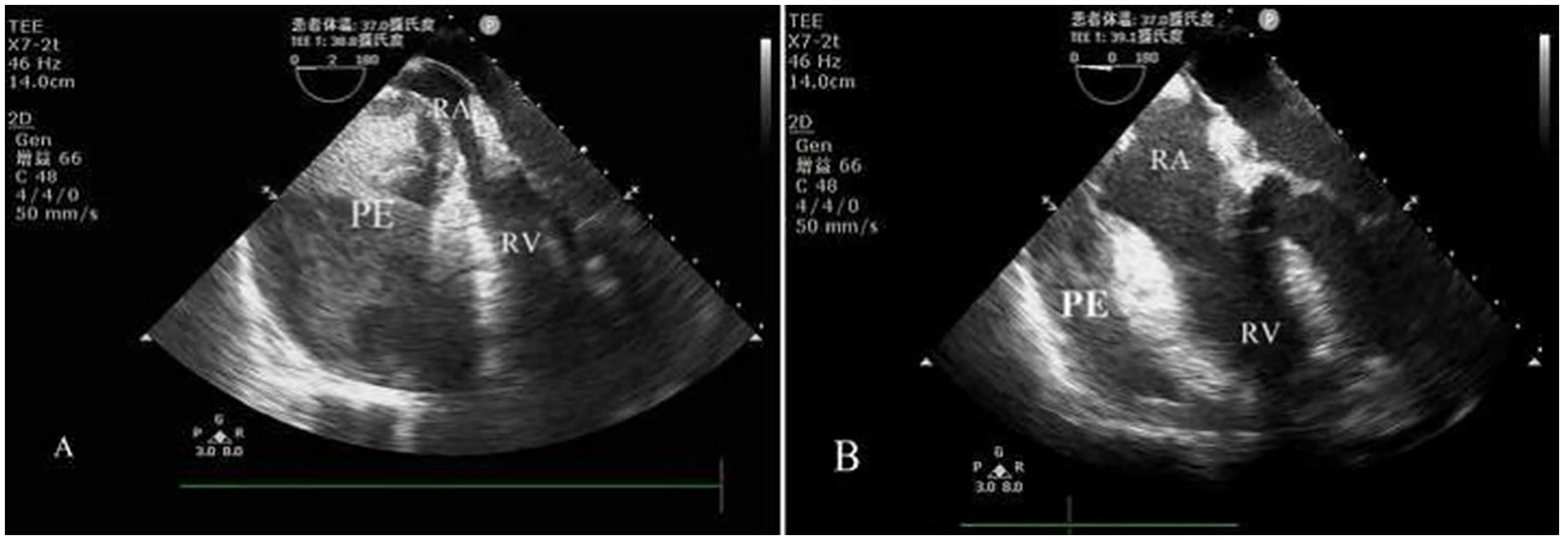
Four-chamber view on transesophageal echocardiography (TEE). **(A)** A large loculated pericardial effusion (PE) and the heart chambers on the right side collapsed, especially the right atrium (RA); RV, right ventricle. **(B)** Pericardial effusion (PE) decreased significantly, and the right atrium (RA) was refilled after subxiphoid surgical pericardial window and drainage; RV, right ventricle.

Immediately after DSA, the ECG and arterial blood pressure wave showed a straight line, and cardiac arrest was confirmed. Chest compressions were commenced immediately, and epinephrine 100 μg was intravenously administered. Spontaneous circulation returned after about 30 s (blood pressure 145/93 mmHg, CVP 21–35 mmHg, and heart rate 145 bpm with atrial fibrillation), and bilateral NIRS remained undetectable. Acute cardiac tamponade was highly suspected, and TEE was performed immediately, showing a large loculated pericardial effusion and right heart chamber collapse, especially the right atrium (Figure 2A). Thoracic and abdominal sonography found no other abnormal bleeding.

Subxiphoid surgical pericardial window and drainage were performed emergently instead of catheter pericardiocentesis: a median incision of approximately 3 cm was made in the xiphoid area, the pericardial parietal layer was bluntly dissected under direct vision, a large amount of bloody fluid was found in the pericardial cavity, and approximately 300 mL of bloody fluid was aspirated with a drainage tube. No obvious active bleeding points were found in the pericardial cavity during the operation. Three minutes after aspiration, there was no sign of further bleeding, and a 16F silicone drainage tube was placed in the pericardial cavity for continuous closed drainage.

TEE reexamination indicated that the pericardial effusion was significantly reduced and the right atrium was refilled (Figure 2B). The patient’s CVP decreased to 24 mmHg, blood pressure increased to 147/74 mmHg, and atrial fibrillation reversed to sinus rhythm automatically. After the diagnosis of acute cardiac tamponade, the following anticoagulation/antiplatelet therapy adjustments were implemented: (1) immediate suspension of all anticoagulant drugs, no additional unfractionated heparin was administered, and protamine (total dose 50 mg, titrated based on ACT monitoring) was used to reverse the residual anticoagulant effect of heparin; (2) the standard dual antiplatelet therapy (aspirin 100 mg qd + clopidogrel 75 mg qd) was delayed until the 7th day after tamponade (after confirmation of no further pericardial bleeding on TEE and stable hemodynamics). For postoperative persistent atrial fibrillation, a conservative strategy of no oral anticoagulation was adopted during hospitalization, considering the high risk of rebleeding; after discharge, the patient was followed up with TEE to evaluate left atrial thrombus, and oral apixaban (5 mg bid) was initiated 4 weeks after discharge (no thrombus was found).

### Perioperative biological data and short-term prognosis

Serial perioperative biological indicators were dynamically monitored, including arterial blood gases, lactate, hemoglobin/hematocrit, and coagulation profiles (Table 1). The patient’s lactate level increased to 4.2 mmol/L after cardiac arrest and gradually decreased to the normal range within 24 h after drainage; hemoglobin/hematocrit showed a mild decrease, but no persistent decline, and no blood transfusion was required. The patient was transferred to the intensive care unit (ICU) for postoperative monitoring, with no abnormal neurological deficits or organ dysfunction, and was discharged from the hospital 2 weeks later with regular follow-up.

### Literature review

We conducted a systematic literature retrieval on pericardial tamponade complicating endovascular aortic procedures in the databases of PubMed, Embase, Web of Science and CNKI (retrieval time: January 2010–December 2024), with the retrieval keywords: “cardiac tamponade,” “endovascular stent grafting”, “aortic dissection”, “TEVAR”, “pericardial effusion” and “vascular intervention.” The inclusion criteria were: (1) clinical case reports, cohort studies, or meta-analyses on pericardial tamponade after endovascular aortic intervention; (2) the study subjects were patients with aortic dissection/aneurysm undergoing TEVAR/EVAR; (3) the full text of the literature was available, and the clinical data were complete. The exclusion criteria were: (1) tamponade caused by non-interventional factors (e.g., traumatic pericardial effusion and primary cardiac disease), (2) review articles without original clinical data, and (3) literature with incomplete data and unable to extract effective information.

### Incidence and common causes

A total of 18 relevant cohort studies and 42 case reports were included in the final analysis. The results showed that the overall incidence of pericardial tamponade complicating endovascular aortic intervention was 0.8–2.3%, and the mortality rate was as high as 35–50% in patients with cardiac arrest caused by tamponade (8, 13, 14). The common causes of tamponade were aortic rupture/perforation (78% of cases), cardiac perforation (12% of cases), and iatrogenic vascular injury (6% of cases) (9, 15). Only 4 case reports (including this case) reported tamponade without arterial/cardiac injury, accounting for about 4% of all cases, and the main suspected causes were perioperative anticoagulation, pericardial adhesion after previous cardiac surgery, and long procedural duration (10−12).

### Clinical manifestations and diagnostic strategies

The clinical manifestations of perioperative cardiac tamponade are non-specific, mainly including progressive hypotension, increased CVP, decreased urine output, and altered consciousness (16). The European Society of Cardiology (ESC) 2021 Pericardial Disease Guidelines noted that TEE is the gold standard for the rapid diagnosis of perioperative cardiac tamponade, with a sensitivity and specificity of >95%, and can realize quantitative assessment of pericardial effusion and dynamic monitoring of cardiac chamber morphology (17). Transthoracic echocardiography (TTE) is the first-line diagnostic method for non-surgical patients, but its diagnostic efficiency is limited in patients with positive-pressure ventilation, surgical dressing, and supine position (18). Focused cardiac ultrasound (FCU) can be used for rapid screening for tamponade in emergency situations, and the typical signs include diastolic right atrial/ventricular collapse, a dilated non-pulsatile inferior vena cava, and a “swinging heart” (19).

### Therapeutic strategies

The core therapeutic principle of perioperative cardiac tamponade is timely drainage of pericardial effusion and reversal of hemodynamic instability (18, 29). Catheter pericardiocentesis is the first-choice intervention for non-surgical patients with small to moderate pericardial effusion and no active bleeding (20). For patients with large pericardial effusions, cardiac arrest, or suspected active bleeding, surgical pericardial window drainage is the preferred method, which can achieve complete drainage of pericardial effusions and exploration of bleeding points under direct vision (21). Subxiphoid surgical pericardial window drainage is the most commonly used surgical approach in perioperative emergency situations, with the advantages of a simple procedure, rapid onset, and reduced trauma (22). In addition, timely adjustment of anticoagulation/antiplatelet therapy is crucial for avoiding rebleeding, including suspension of anticoagulants, reversal of heparin effect with protamine, and delayed initiation of antiplatelet therapy (23).

### Comparison with the present case

The present case is consistent with the clinical characteristics of rare tamponade without arterial/cardiac injury reported in previous literature, including a history of cardiac surgery, long procedural duration, and systemic heparin anticoagulation (10, 11). The uniqueness of this case lies in the occurrence of protamine anaphylactic reaction before tamponade, which synergistically aggravated the hemodynamic disorder and masked the early manifestations of tamponade, leading to delayed recognition. In addition, the patient achieved rapid recovery after emergency subxiphoid surgical pericardial window drainage, which further confirmed the effectiveness of this intervention for tamponade-induced cardiac arrest in perioperative settings. This case enriches the clinical spectrum of pericardial tamponade complicating endovascular aortic procedures and provides new clinical evidence for the correlation between protamine reaction and tamponade.

## Discussion

### Etiological analysis of cardiac tamponade: exclusion, evidence-based inference, and self-limited confirmation

In this case, the cause of cardiac tamponade was rigorously analyzed by combining clinical data, procedural characteristics, imaging results, and postoperative follow-up, and a structured differential discussion was conducted to exclude definite etiologies and infer possible causes from clinical evidence.

First, definite etiologies were completely excluded: (1) Aortic injury and cardiac perforation were excluded because DSA reexamination confirmed the normal position and patency of the stent, no aortic rupture/perforation or cardiac perforation was found; (2) Iatrogenic vascular injury was excluded because thoracic and abdominal sonography found no abnormal bleeding in other parts, and no active bleeding points were found during surgical pericardial window drainage; (3) Traumatic pericardial effusion was excluded because the surgical operation was performed under standard aseptic conditions and no iatrogenic trauma to the pericardium was found during the procedure.

Then, possible etiologies were inferred with clinical evidence: The tamponade was most likely caused by microvascular oozing in the pericardium combined with mild bleeding at the pericardial adhesion site after previous cardiac surgery, and the inducing factors included: (1) systemic heparin anticoagulation: The patient received a large dose of unfractionated heparin (150 IU/kg) with ACT maintained at 375–999 s, which significantly reduced the blood coagulation function and increased the risk of microvascular bleeding; (2) long procedural duration: the entire second-stage operation lasted for 7 h, and the long-term surgical stimulation may lead to increased vascular permeability of the pericardium; (3) previous cardiac surgery history: The patient underwent hemi-arch replacement and Bentall operation 20 days before, and pericardial adhesion was formed after surgery, which made the microvascular at the adhesion site more prone to bleeding under the action of anticoagulation; (4) protamine reaction: The histamine release caused by protamine anaphylactic reaction further increased the vascular permeability of the pericardium and promoted microvascular oozing.

Finally, the self-limited mechanism of bleeding was confirmed: No obvious active bleeding points were found during surgical drainage, and no further bleeding was found after 3 min of aspiration; the patient’s hemoglobin/hematocrit only showed a mild decrease and no persistent decline, and no blood transfusion was required (30); TEE reexamination at 24 h after drainage showed no recurrent pericardial effusion. The above evidence confirmed that bleeding was self-limited microvascular oozing and no persistent bleeding source was present in the pericardial cavity.

### Correlation between the protamine anaphylactic reaction and cardiac tamponade

Protamine anaphylactic reaction is not only an independent cause of perioperative hemodynamic instability but also has a significant synergistic effect on cardiac tamponade, and its interference with the early diagnosis of tamponade is an important clinical problem that needs to be vigilant. This section discusses the correlation between the two from three aspects: pathophysiological effects, synergistic effect, and diagnostic interference.

1. Pathophysiological effects of protamine anaphylactic reaction: Protamine is a positively charged polypeptide that can cause an anaphylactic reaction by activating the complement system and releasing histamine, leukotrienes, and other inflammatory mediators (12). The main pathophysiological effects include: (1) systemic hemodynamic instability: peripheral vasodilation and decreased cardiac contractility lead to acute hypotension and tachycardia; (2) pulmonary vasoconstriction and right ventricular failure: pulmonary vasospasm increases pulmonary vascular resistance, leading to right ventricular overload and failure; (3) increased vascular permeability: inflammatory mediators release leads to increased vascular endothelial permeability, which promotes microvascular oozing in tissues and organs including the pericardium.
2. Synergistic effect of protamine reaction and cardiac tamponade on hemodynamic disorder: The protamine reaction in this case occurred before the onset of tamponade, and the two formed a significant synergistic effect to aggravate the patient’s hemodynamic disorder: (1) The right ventricular failure caused by protamine reaction increased the right heart filling pressure, which further aggravated the hemodynamic disorder caused by pericardial effusion and right heart chamber collapse; (2) The increased vascular permeability caused by protamine reaction promoted pericardial microvascular oozing and accelerated the formation and development of pericardial effusion; (3) The hypotension caused by protamine reaction required vasopressor drugs for maintenance, which masked the progressive hypotension caused by early tamponade and delayed the intervention time.
3. Interference of protamine reaction on early diagnosis of cardiac tamponade: The clinical manifestations of protamine reaction (hypotension, tachycardia, increased CVP) are highly consistent with the early manifestations of cardiac tamponade, which leads to the confusion of clinical diagnosis (24). In this case, the initial hemodynamic instability was attributed to a protamine reaction, and the progressive increase in CVP and decrease in NIRS were considered as the sequelae of the protamine reaction, ignoring the possibility of cardiac tamponade. This diagnostic interference led to the delayed performance of TEE and the occurrence of cardiac arrest, which is a valuable clinical lesson. For patients with persistent hemodynamic instability after a protamine reaction, TEE should be performed as soon as possible to exclude cardiac tamponade, regardless of whether there are obvious signs of aortic/cardiac injury.

### Clinical limitations of the case

The main clinical limitation of this case is the lack of targeted TEE/TTE monitoring during the interval between the two-stage surgeries. Although CTA was performed on the 14th postoperative day and no obvious dissection progression or pericardial effusion was found, the lack of dynamic echocardiographic monitoring made it impossible to detect potential asymptomatic mild pericardial effusion. For patients undergoing staged aortic dissection surgery with a history of cardiac surgery, regular echocardiographic monitoring during the interval is recommended to detect early pericardial effusion and avoid the development of severe tamponade. In addition, thromboelastography (TEG) was not performed during the perioperative period, which limited the comprehensive evaluation of the patient’s coagulation function and individualized adjustment of anticoagulation therapy. TEG can dynamically monitor the whole process of blood coagulation and fibrinolysis, and is more suitable for guiding perioperative anticoagulation management in vascular surgery patients (25, 26).

### Perioperative anticoagulation management for tamponade after endovascular intervention

Individualized adjustment of anticoagulation/antiplatelet therapy is crucial for the treatment of tamponade after endovascular aortic intervention with systemic anticoagulation (23). In this case, we adopted a step-by-step anticoagulation adjustment strategy: (1) immediate suspension of all anticoagulant drugs to avoid further bleeding; (2) titrated use of protamine to reverse the residual anticoagulant effect of heparin based on ACT monitoring, avoiding excessive protamine use that may lead to coagulation dysfunction; (3) delayed initiation of dual antiplatelet therapy until the 7th day after tamponade, after confirmation of no further pericardial bleeding on TEE; (4) conservative anticoagulation strategy for postoperative atrial fibrillation during hospitalization, and oral factor Xa inhibitor (apixaban) was initiated after discharge with TEE confirmation of no left atrial thrombus. This strategy balances the risks of rebleeding and stent thrombosis/atrial fibrillation thromboembolism, and is suitable for patients with tamponade without arterial/cardiac injury after endovascular intervention.

## Conclusion

This case reports a rare case of cardiac arrest due to acute cardiac tamponade without aortic/cardiac injury in a patient undergoing second-stage TEVAR for DeBakey type I aortic dissection after protamine anaphylactic reaction, and supplements a systematic literature review on pericardial tamponade complicating endovascular aortic procedures. Based on the clinical characteristics of this case and the results of the literature review, we put forward the following specific and targeted clinical take-home messages, which provide valuable guidance for the perioperative management of staged aortic dissection surgery:

1. For patients undergoing staged TEVAR for DeBakey type I aortic dissection with a history of cardiac surgery, long procedural duration, and systemic anticoagulation, pericardial tamponade should be highly vigilant even in the absence of obvious aortic/cardiac injury. Microvascular oozing in the pericardium, combined with pericardial adhesion bleeding, is the main suspected cause, and timely TEE examination is the key to early diagnosis.
2. Protamine anaphylactic reaction can synergize with cardiac tamponade to aggravate perioperative hemodynamic disorder and mask the early clinical manifestations of tamponade. For patients with persistent hemodynamic instability after protamine reaction, TEE should be performed as soon as possible to exclude cardiac tamponade, and multi-modal monitoring (including NIRS, CVP, and serial lactate) should be enhanced to identify early abnormal changes.
3. Transesophageal echocardiography (TEE) is the gold standard for the rapid diagnosis of perioperative cardiac tamponade, and timely subxiphoid surgical pericardial window and drainage is an effective intervention measure for tamponade-induced cardiac arrest. This surgical approach has the advantages of simple operation, rapid onset, and direct vision exploration, and is the first choice for emergency treatment of severe perioperative tamponade.
4. For staged aortic dissection surgery, complete interval imaging evaluation (including CTA and echocardiography) and comprehensive perioperative biological/physiological index monitoring are the key to early identification of severe complications and improvement of surgical safety. Individualized adjustment of anticoagulation/antiplatelet therapy based on the patient’s coagulation function and bleeding status is crucial for avoiding rebleeding and improving the prognosis of patients with tamponade.
5. Perioperative management of aortic dissection surgery should pay attention to the correlation between various adverse events and avoid a single-factor diagnosis for persistent hemodynamic instability. A comprehensive diagnostic approach that integrates clinical manifestations, imaging examinations, and laboratory indicators is the basis for accurate and timely diagnosis of severe perioperative complications.

## Data Availability

The original contributions presented in the study are included in the article/supplementary material, further inquiries can be directed to the corresponding author.
